# A Genome-Wide Association Study of Circulating Serum Choline, Betaine, Dimethylglycine, and Their Ratios

**DOI:** 10.3390/nu17162630

**Published:** 2025-08-14

**Authors:** Lauren E. Louck, Kevin C. Klatt, Taylor C. Wallace, Jiantao Ma, Mei Chung

**Affiliations:** 1 Friedman School of Nutrition Science and Policy, Tufts University, Boston, MA 02111, USAtaylor.wallace@me.com (T.C.W.); jiantao.ma@tufts.edu (J.M.); 2 Nutritional Sciences and Toxicology, University of California, Berkeley, CA 94720, USA; klatt@berkeley.edu; 3 School of Medicine and Health Sciences, George Washington University, Washington, DC 20037, USA; 4 Think Healthy Group, LLC, Washington, DC 20001, USA

**Keywords:** choline, betaine, dimethylglycine, genome-wide association study, genetic variation

## Abstract

*Background/Objectives:* Genetic variation has been thought to alter the human dietary requirement for choline and subsequent circulating levels of its metabolites betaine and dimethylglycine (DMG). The aim of this genome-wide association study (GWAS) was to identify single nucleotide polymorphisms (SNPs) associated with serum choline, betaine, and dimethylglycine (DMG) as well as choline-to-betaine and betaine-to-DMG ratios. *Methods:* Data from the Collaborative Study of Genes, Nutrients and Metabolites (CSGNM; *n* = 2402) were used to model individual associations of choline, betaine, and DMG circulating metabolites and their ratios with 680,975 SNPs, using linear regression. Models were unadjusted (model 1), adjusted for age and sex (model 2), and further adjusted for selected principal components (model 3) and B_12_, B_9_, B_6_, and holotranscobalamin (model 4). Statistical significance was set at *p* < 5.0 × 10^−5^. Affected SNPs in the dbSNP (database of Single Nucleotide Polymorphisms) were then identified. *Results:* GWAS revealed both intuitive and novel results, including the recently described SLC25A48, several intronic variants in the gene encoding LYPLAL1, and a pair of SNPs present in the intronic region of PID1 related to serum choline. SNPs related to betaine and DMG included SLCA12, BHMT, DMGDH, and additional SLC family transporters that require further validation. While exploratory, GWAS of the choline-to-betaine and betaine-to-DMG ratios revealed common targets with direct links to choline and one-carbon metabolism. *Conclusions:* These results suggest that metabolic handling of choline has genetic determinants not formerly recognized in the scientific literature. Replication is needed in larger cohorts due to low statistical power.

## 1. Introduction

An adequate supply of choline is essential for supporting its diverse physiological functions, including maintenance of hepatic, cardiovascular, and neurological function [1,2]. Choline is a critical metabolic substrate for the synthesis of the following: phosphatidylcholine, a key component of membranes; lipoproteins, cell signaling compounds; acetylcholine, a critical neurotransmitter; and betaine, an osmolyte and methyl donor that supports one-carbon metabolism. Choline’s oxidation to betaine and use as a methyl donor by betaine-homocysteine methyltransferase (BHMT) regenerates methionine from homocysteine, yielding dimethylglycine (DMG), which can be further oxidatively demethylated and contribute one-carbon units to folate for use in one-carbon metabolism to support methylation reactions as well as the synthesis of nucleotides.

Maintenance of an adequate supply of choline to tissues is supported by the endogenous synthesis of choline through the phosphatidylethanolamine N-methyltransferase pathway, but it is insufficient to maintain plasma choline and betaine levels and organ function without an exogenous source of choline in the diet. When dietary choline intakes are adequate, plasma levels of choline, betaine, and DMG can still vary considerably among healthy individuals (7–20 μM, 20–60 μM, and 2.5–7.5 μM, respectively) [3]. In general, dietary intakes of choline and betaine only minimally impact plasma levels [4], suggesting other biological factors are likely to influence metabolite homeostasis. To date, little is known about variation in metabolic kinetics that might influence plasma metabolite concentrations, including variability in choline and betaine absorption, altered distribution of metabolites between plasma and tissue compartments, altered metabolism, and/or altered excretion.

It is thought that genetic factors may contribute to the metabolic homeostasis of choline, betaine, and DMG. Several common single nucleotide polymorphisms (SNPs) in genes encoding enzymes involved in choline and one-carbon metabolism have been thought to significantly modify the odds of developing organ dysfunction under conditions of deficiency [5]. However, this body of research comprises predominantly post hoc analyses of smaller trials that have employed a candidate gene approach, and variants in these candidate genes explain only a limited degree of the variation in circulating metabolite concentrations, suggestive of untapped genetic determinants.

The emergence of studies with high-throughput genomic data as well as metabolite analysis have allowed for the discovery of novel genetic determinants of circulating metabolites. These cohorts facilitate the discovery of SNPs that influence plasma metabolite levels and additionally have the potential to uncover novel proteins involved in metabolic homeostasis. Indeed, recent genome-wide association studies (GWAS) assessing plasma choline levels have uncovered candidate transporters for the ubiquitously expressed plasma membrane transporter not previously associated with choline dynamics [6]. Candidate SNPs identified in these analyses also serve as potential modifiers of nutrient intake requirements, broadening the rationale for precision nutrition investigations. While GWAS hold great potential, results from our systematic review of genetic determinants of 42 circulating choline-related metabolites, primarily trimethylamine N-oxide (TMAO), betaine, sphingomyelins, lysophosphatidylcholines, and phosphatidylcholines, identified a need to undertake GWAS analyses in well-phenotyped cohorts that assessed choline and its metabolites using targeted, absolute quantification (as opposed to the untargeted, relative quantification values common to metabolomic data) [7]. To this end, we have undertaken a GWAS of serum choline, betaine, and DMG utilizing data from the Collaborative Study of Genes, Nutrients and Metabolites (CSGNM) to expand on the genetic determinants of circulating metabolite concentrations and facilitate the discovery of candidates for functional analyses.

## 2. Materials and Methods

### 2.1. Study Sample

The CSGNM conducted genome-wide genotyping and serum metabolite quantification on 2402 ethnically Irish participants attending Trinity College Dublin, who were recruited between February 2003 and 2004 [8]. Participants provided a non-fasting blood sample for genetic and circulating metabolite quantification. Extracted DNA was genotyped using the HumanOmni1-Quad BeadChip (Illumina, San Diego, CA, USA). Circulating choline, betaine, and DMG were reported in micromoles per liter. Participants also completed a diet, lifestyle, and demographics questionnaire, which included information on age, sex, height, weight, medical conditions, smoking, dietary habits, and consumption of alcohol, fortified foods, and dietary supplements. Participants with non-Irish grandparents, major medical problems, or missing questionnaires were excluded from the cohort. We requested the CSGNM data for the present analyses from the dbGaP (database of Genotypes and Phenotypes) (*n* = 2232) [9]. The study has been described in detail elsewhere [8].

### 2.2. Genetic Quality Control

Genetic data quality control was conducted using PLINK (version 1.9) [10]. SNPs were limited to autosomal chromosomes. SNPs and individuals with a >20% missingness rate, call rate < 98%, minor allele frequency (MAF) < 0.05, or Hardy–Weinberg equilibrium *p* < 1 × 10^−6^ were excluded from our analyses. Sex discrepancies were checked; males with a homozygosity estimate < 0.08 and females with a homozygosity estimate > 0.2 were excluded from our analyses. Relatedness was investigated by calculating identity by descent of all sample pairs. If pi-hat was >0.2, the related pairs were excluded from our analyses. Individuals who deviated more than 3 SD from the heterozygosity rate mean were additionally excluded from our analyses. The 1000 Genomes Project data served as a reference to help define genetic ancestry [11]. Multidimensional scaling (MDS) analysis was conducted using PLINK to evaluate population structure and identify ethnic outliers by anchoring individual genetic data to the 1000 Genomes reference panel. Genotype data from CSGNM and 1000 Genomes were merged after harmonizing SNP sets, aligning reference alleles, and resolving strand inconsistencies. A pairwise identity-by-state (IBS) distance matrix was computed from linkage disequilibrium-pruned SNPs. An MDS plot was created using RStudio. We then performed MDS using only the Irish samples after excluding any potential ethnic outliers. The top 10 MDS components derived from the Irish samples were then extracted. To determine component inclusion, Scree and Scree-Eigen plots were created using the factoextra R package (version 1.0.7). Due to the small sample size, imputation and fine mapping were not performed in these analyses.

### 2.3. Genome-Wide Association and Statistical Analysis

The distribution of serum choline was visualized with a histogram and QQ plot and quantitatively tested using the Shapiro–Wilk test. Genome-wide association analysis was performed using linear regression models to test the individual association of serum choline, betaine, and DMG as well as choline-to-betaine and betaine-to-DMG ratio concentrations (in micromoles per liter) with 680,975 SNPs, under an additive genetic model. Serum choline models were unadjusted (model 1), adjusted for age and sex (model 2), and further adjusted for selected principal components (PCs) (model 3) and B_12_, B_9_, B_6_, and holotranscobalamin (HoloTC) (model 4). Models 3 and 4 were employed for serum betaine, DMG, and choline-to-betaine and betaine-to-DMG ratios. λ values were assessed in all choline-to-betaine and betaine-to-DMG ratio models. The analysis was conducted in R (version 4.2.0; R Foundation for Statistical Computing) on the Tufts University secure high-performance computing cluster.

QQ plots were used to visualize deviations of the observed *p*-values for each SNP under the null hypothesis. *p* < 5.0 × 10^−5^ was used as the genome-wide statistical significance threshold. False discovery rate (FDR) adjustments were performed for comparison using 0.05 as the significance threshold. Significant SNPs were searched for in the National Center for Biotechnology Information dbSNP (database of Single Nucleotide Polymorphisms) [12] to identify the associated gene. LocusZoom [13] was used to create a plot displaying association signals, linkage disequilibrium, and nearby genes for relevant loci from choline model 3. Significant SNPs from models 3 and 4 were searched on the National Institutes of Health Genotype-Tissue Expression (GTEx) Portal [14] to determine whether SNPs were significantly associated with altered gene expression levels in tissues. A final *p* < 5.0 × 10^−5^ was used as the genome-wide significance threshold. FDR correction was also applied as an additional method and used to compare against SNPs meeting the *p* < 5.0 × 10^−5^ threshold, using an FDR threshold of < 0.05. Finally, we compared SNPs identified in our GWAS with those within our previously published scoping review [7], which sought to identify and summarize reported SNPs present in the scientific literature prior to these analyses.

## 3. Results

### 3.1. Participant Characteristics

**Figure 1:**
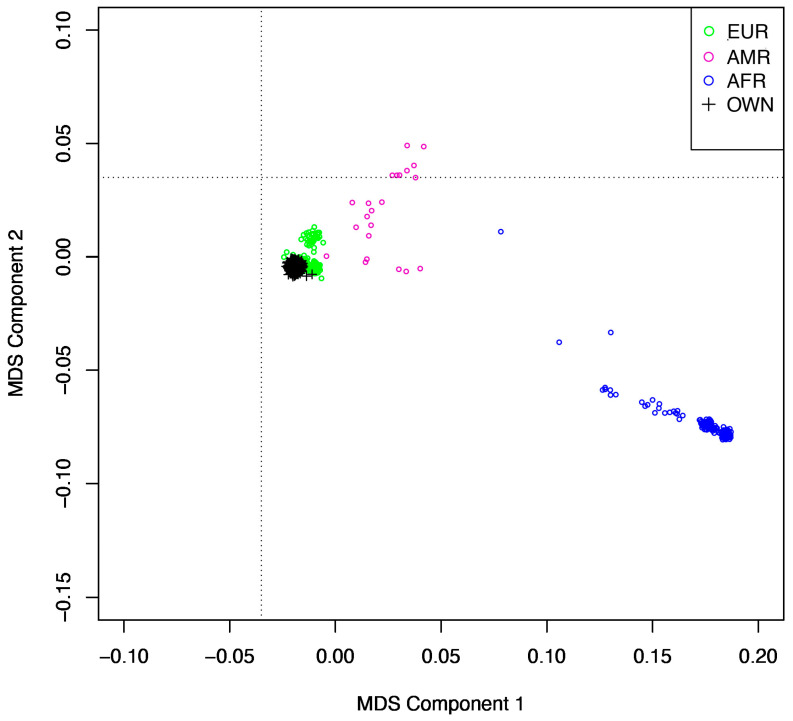
Multidimensional scaling plot of the study cohort anchored to the 1000 Genomes reference panel. AFR = African ancestry; AMR = Admixed American ancestry; EUR = European ancestry; OWN = the study cohort.

A summary of participant characteristics is displayed in Table 1. The total genotyping rate was 0.99. All participants passed the 98% call rate. We removed 3095 variants with a missingness rate > 20%, 71,983 variants with a MAF < 0.05, and 1524 variants with a Hardy–Weinberg equilibrium *p* < 1 × 10^−6^ from the dataset. Thus, there are a total of 604,373 SNPs included in our analyses. Since genetic data on participant sex were not available via dbGaP, phenotypic data were utilized in the analyses. No related individuals with a pi-hat value > 0.02 were identified in the dataset. We removed 12 participants from the dataset (8 with a heterozygosity rate deviating >3 SD from the mean and 4 with missing serum choline data). An MDS plot anchored to 1000 Genomes reference data is presented in Figure 1. For component inclusion, according to the Scree plot, there was a noticeable inflection point at component 4. The Scree-Eigen plot showed the first five components had eigenvalues > 1. Thus, components 1 to 4 were included as covariates based on information from the Scree (Figure S1) and Scree-Eigen (Figure S2) plots.

### 3.2. Metabolite Distributions

Based on histogram, QQ plot, and Shapiro–Wilk test results for serum choline (W = 0.96, *p* < 2.2 × 10^−16^), betaine (W = 0.98, *p* < 2.2 × 10^−16^), and DMG (W = 0.90, *p* < 2.2 × 10^−16^), the distribution of each metabolite was right skewed. Therefore, log-transformations were applied to normalize the distributions.

### 3.3. GWAS of Serum Choline, Betaine, and DMG

**Figure 2:**
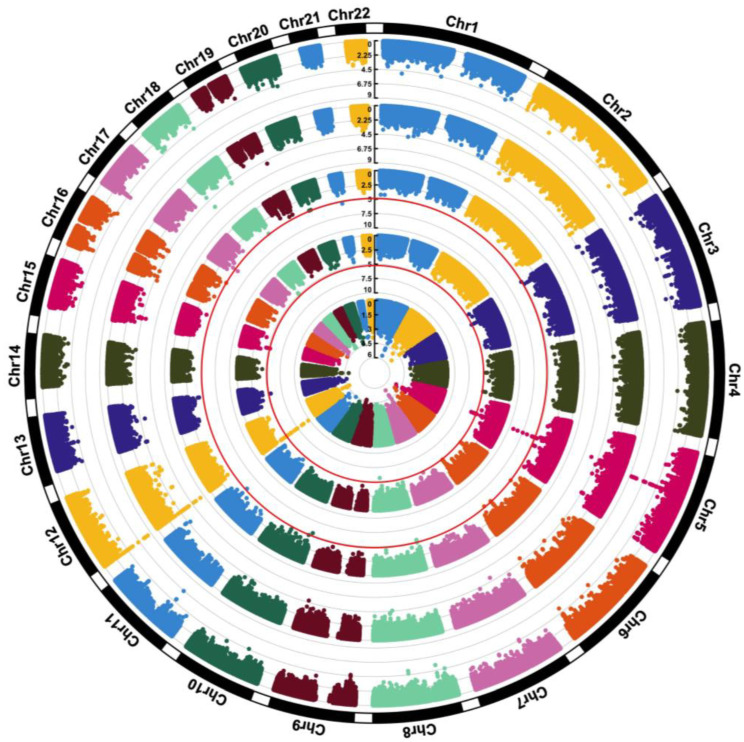
A circular Manhattan plot visualizing all model 3 results (adjusted for age, sex, and PCs). Starting from the inner circle, the rings represent choline, betaine, DMG, and the choline-to-betaine and betaine-to-DMG ratios. The scales represent GWAS *p*-values, individual data points represent SNPs, and SNPs are aggregated by chromosome. Red circular lines mark the significance *p* value threshold.

**Figure 3:**
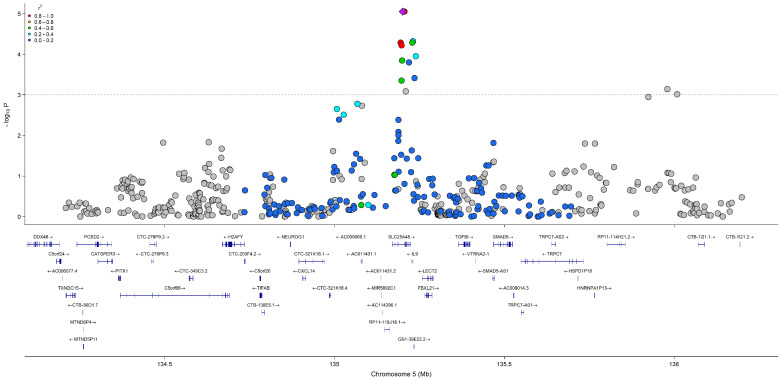
LocusZoom plot of chromosome 5 from the choline GWAS (model 3), displaying association signals, linkage disequilibrium, and nearby genes. The purple diamond indicates the most significant SNP, and gray solid circles indicate SNPs not in NIH LDlink API database.

All SNP and regression results from model 3 that met the *p* < 5.0 × 10^−5^ significance threshold are presented in Table 2, Table 3 and Table 4. Model 4 results and FDR comparisons are presented in Tables S1–S3. QQ plots used to visualize deviations of the observed *p*-values for each SNP under the null hypothesis are presented in Figures S3, S4 and S5 for choline, betaine, and DMG (model 3), respectively. QQ plots for choline, betaine, and DMG (model 4) are presented in Tables S6, S7 and S8, respectively. All results from model 3 are visualized in the circular Manhattan plot presented in Figure 2. Model 3 results for SNPs in choline and folate metabolism that have previously been identified to modify the risk of organ dysfunction in post hoc analyses of controlled feeding studies that administer choline-deficient diets (<50 mg/d) to participants are presented in Table 5. None of these SNPs reached statistical significance (*p* < 5.0 × 10^−5^). A LocusZoom plot of chromosome 5 from the choline model results is presented in Figure 3. Data from GTEx that matched genes from SNP IDs meeting the significance threshold for model 3 are presented in Tables S7, S8, S10, S11, S13 and S14.

### 3.4. GWAS of Serum Choline-to-Betaine and Betaine-to-DMG Ratios

Both choline-to-betaine and betaine-to-DMG ratio distributions were right skewed. Therefore, for clarity of the interpretation of results, genomic control (*λ*) values were checked for both the choline-to-betaine (model 3: *λ* = 0.982; model 4: *λ* = 0.97) and betaine-to-DMG (model 3: *λ* = 1.01; model 4: *λ* = 1.01) ratio models. No transformations were performed, since *λ* values were deemed acceptable for both ratio models. SNP and regression results from model 3 with a *p* < 5.0 × 10^−5^ significance threshold for the choline-to-betaine and betaine-to-DMG ratio models are presented in Table 6 and Table 7, respectively. Model 4 results and FDR comparisons are presented in Tables S4 and S5. QQ plots used to visualize deviations of the observed *p*-values for each SNP under the null hypothesis are presented in Figures S9 and S10 for choline-to-betaine and betaine-to-DMG ratios (model 3), respectively. QQ plots for choline-to-betaine and betaine-to-DMG ratios (model 4) are presented in Tables S11 and S12, respectively. Data from GTEx that matched genes from SNP IDs meeting the significance threshold for model 3 are presented in Tables S18 and S19.

### 3.5. SNP Literature Comparison

In our recent scoping review, we identified 577 SNPs from prior GWAS of circulating choline metabolites, of which only 28 were identified in ≥2 unique cohorts. None of these SNPs previously identified in the scientific literature met our *p* < 5.0 × 10^−5^ significance threshold; however, 2 SNPS (rs1800588 and rs261332) reached the *p* < 5.0 × 10^−3^ threshold.

## 4. Discussion

Although wide variability in circulating choline, betaine, and DMG is observed in generally healthy populations, determinants of this variation are poorly characterized in the scientific literature to date. The results of this GWAS indicate the potential for significant genetic determinants of these key metabolites. While our results certainly require repetition in larger cohorts, they are useful as candidate genes for functional analyses to uncover novel cellular determinants of choline handling and metabolism and for future exploration in diet-x-genotype analyses aiming to advance ‘precision nutrition’.

The idea that genetic variants modify choline metabolism is generally supported by post hoc analyses of controlled feeding trials demonstrating that when individuals are fed choline-deficient diets, the carrier status for common SNPs in choline and folate metabolism dramatically modifies the risk of displaying choline deficiency–associated organ dysfunction [5,15]. Whether such genetic variants play a role in explaining the variation in circulating choline metabolites in the general population has received limited attention, in part due to the lack of large cohorts with complete genetic and metabolite data. In our analysis, classical variants described as modifiers of choline deficiency–associated phenotypes did not emerge as determinants of plasma choline (Table 5). Rather, SNPs nearby to genes not previously linked directly to choline metabolism emerged in our analysis, with the exception of the recently described solute carrier SLC25A48 locus that encodes the mitochondrial choline importer [16,17].

Several of these novel SNPs not previously linked to choline homeostasis, particularly those with multiple SNPs, may be worthy of additional functional characterization. While the emergence of *SLC25A48* in this analysis is encouraging, we note caution in assuming observed relationships represent causal links to choline biology. Several intronic variants in the gene encoding lysophospholipase 1 (LYPLAL1), a cytosolic acyl thioesterase protein reported to de-palmitoylate its target proteins, were associated with serum choline concentrations; intriguingly, LYPLAL1 has been reported to lack lysophospholipase activity, a potential avenue toward a more direct link to choline homeostasis through lysophosphatidylcholine metabolism. LYPLAL1 has not previously been associated directly with choline metabolism; given its broad tissue expression profile and the limited description of its target proteins for de-palmitoylation, its link to choline metabolism remains speculative. Variants in LYPLAL1 have been associated with other metabolic phenotypes, including waist-to-hip ratio, fat distribution, and metabolic dysfunction-associated steatotic liver disease (MASLD; previously nonalcoholic fatty liver disease or NAFLD) [18,19,20,21,22,23], and results in sex-specific body composition phenotypes in LYPLAL1 knockout mice, which suggests that this protein’s de-palmitoylation activity has broad effects on metabolic homeostasis. Consistent with such sex-specific effects in the knockout mouse, an indirect link between LYPLAL1 and choline homeostasis may result from differences in estrogen signaling secondary to altered palmitoylation of its receptor [24]. An additional pair of SNPs present in the intronic region of phosphotyrosine interaction domain containing 1 (PID1) emerged in our analysis that have also been reported to regulate key metabolic processes, including the clearance of triglyceride-rich lipoproteins as well as antagonizing insulin receptor signaling, and common variants in PID1 are associated with obesity and cardiometabolic disease. Direct links between PID1 and choline metabolism have also not been reported but might theoretically occur due to altered lipoprotein fluxes and redistribution of choline across metabolic compartments.

Our GWAS results for serum betaine and DMG, the oxidative products of choline metabolism that shunt choline toward one-carbon metabolism, revealed both intuitive and novel results worthy of exploration in repetition cohorts and for further functional validation. Top hits directly related to betaine and DMG metabolism included the following: SLC6A12, the known sodium- and chloride-dependent betaine transporter; BHMT, catalyzing the use of betaine as a methyl donor to remethylate homocysteine; and dimethylglycine dehydrogenase (DMGDH), catalyzing the use of DMG as a methyl donor in mitochondrial folate metabolism. While directly linked to choline and one-carbon metabolism, it is intriguing that few variants in these genes have yet been explored as potential modifiers of the dietary choline requirement or in the heterogeneous metabolic responses to choline and betaine supplementation. Additional SLC family transporters also emerged from our analysis, including SLC6A13 and SLC22A3, that require further functional validation for links to one-carbon metabolism.

In addition to absolute concentrations of metabolites, we explored novel ratio outcome variables (choline-to-betaine and betaine-to-DMG) given their direct metabolic interrelationships and potential to inform upon metabolic partitioning. Ratios of choline metabolites have previously been explored in small, randomized trials as well as epidemiological cohorts, but they have received little attention in GWAS investigations. While exploratory, it is notable that this approach revealed common targets, including a broader array of SLC6A12 variants, and a novel lipid droplet-associated phosphatidylserine hydrolase (LDAH), of theoretical relevance given phosphatidylcholine’s role as a precursor to phosphatidylserine. Few other direct links to choline and one-carbon metabolism emerged, questioning the added benefit of this approach; further exploration in larger cohorts is warranted.

Our results have notable strengths and limitations worth highlighting. Our results build on the emerging analyses of cohorts with both whole-genome sequencing as well as metabolomic data, facilitating the uncovering of genetic determinants of metabolic homeostasis and potentially novel genes involved in metabolic regulation. The use of CSGNM data is particularly useful due to the targeted absolute quantification of several nutritional biomarkers, whereas other cohorts use metabolomic data that have semi-quantitative analyses (e.g., untargeted NMR metabolomics). We sought to leverage these strengths to undertake an exploratory GWAS that could consider not only predictors of absolute serum concentrations but also adjust for nutrient status indicators, a critical consideration given the many nutrient–nutrient interactions across choline and one-carbon metabolism. Our analyses, however, have significant limitations, largely owing to the relatively small sample size of the CSGNM cohort and resulting limited statistical power within our analyses. The use of a *p*-value threshold of 5.0 × 10^−5^, which is common in exploratory settings, increases the risk of false positives. While our analysis contained important internal positive controls, such as our observation that variants in SLC25A48, a recently recognized mitochondrial choline importer, are associated with circulating serum choline, betaine, DMG, choline-to-betaine, and/or betaine-to-DMG, not all variants observed are necessarily true genetic determinants and may indeed be false positives. The LD of the region could be indicated by a different gene located close to the signal. The wide array of targets without clear or direct links to choline or general metabolic processes may yield potential insights into cellular determinants of choline homeostasis, but they should generally be viewed with caution until repetition in larger cohorts provide similar signals for these loci and/or other plausible physiological links emerge. Furthermore, the generalizability of our results may be limited by changing in lifestyle, environment, or medical advances over time, given the CSGNM cohort data are 20 years old. We further note that the CSGNM cohort is comprised solely of individuals of Caucasian descent, which inhibits broad extrapolation of the results. Future research should replicate or validate our findings using more recent data.

## 5. Conclusions

In summary, the results of this GWAS suggest that metabolic handling of choline has genetic determinants not formerly recognized in the scientific literature. However, replication is needed in larger cohorts due to low statistical power.

## Figures and Tables

**Table 1:** Participant characteristics.

Variable	Value (*n* = 2220)
Age (y), mean ± SD (min, max)	22.4 ± 1.69 (19.0, 28.0)
Sex, No. (%)	
Male	918 (41.4)
Female	1302 (58.6)
Serum choline (µmol/L), mean ± SD (min, max)	7.65 ± 1.81 (3.30, 19.7)

**Table 2:** GWAS of serum choline (model 3).

SNP	Log (β)	SE	*p*-Value	Gene
rs10779349	0.0141	0.00327	1.63 × 10^−5^	LYPLAL1: intron variant
rs4846551	0.0165	0.00373	1.08 × 10^−5^	LYPLAL1: intron variant
rs6671991	0.0182	0.00385	2.40 × 10^−6^	LYPLAL1: intron variant
rs10205941	−0.0140	0.00322	1.57 × 10^−5^	None
rs6890039	−0.0250	0.00561	8.96 × 10^−6^	SLC25A48: intron variant
rs6596270	−0.0250	0.00561	8.96 × 10^−6^	SLC25A48: synonymous variant
rs4272127	−0.0169	0.00415	4.79 × 10^−5^	SLC25A48: intron variant
rs6601167	0.0228	0.00522	1.26 × 10^−5^	None
rs226915	−0.0229	0.00517	9.97 × 10^−6^	LOC100507336: intron variant
rs2261033	0.0171	0.00399	2.00 × 10^−5^	PRRC2A: intron variant
rs7812117	0.0170	0.00417	4.57 × 10^−5^	NXPH1: intron variant
rs1050290	−0.0162	0.00371	1.40 × 10^−5^	MEOX2: 5′-UTR variant LINC02587: 2KB upstream variant
rs740566	−0.0167	0.00360	3.57 × 10^−6^	LINC02587: 2KB upstream variant MEOX2: 2KB upstream variant
rs12669299	−0.0152	0.00356	2.03 × 10^−5^	None
rs2155939	−0.0189	0.00464	4.81 × 10^−5^	NCALD: intron variant
rs10819341	0.0264	0.00620	2.11 × 10^−5^	None
rs882895	0.0251	0.00606	3.55 × 10^−5^	None
rs7944388	−0.0522	0.0126	3.82 × 10^−5^	None
rs1178704	0.0455	0.0111	4.31 × 10^−5^	PTPRQ: intron variant LOC105369867: intron variant
rs12813355	−0.0165	0.00388	2.21 × 10^−5^	LOC105369867: intron variant
rs149583	0.0247	0.00584	2.48 × 10^−5^	ATXN8OS: intron variant
rs4904895	0.0167	0.00383	1.30 × 10^−5^	LRFN5: intron variant
rs1227005	−0.0207	0.00449	4.08 × 10^−6^	PID1: intron variant
rs1362243	−0.0161	0.00392	4.21 × 10^−5^	PID1: intron variant

Model 3 adjusted for age, sex, and PCs.

**Table 3:** GWAS of serum betaine (model 3).

SNP	Log (β)	SE	*p*-Value	Gene
rs10001408	−0.0321737	0.00786715	4.48 × 10^−5^	TMEM150C: intron variant
rs10016201	−0.0336097	0.00796971	2.57 × 10^−5^	TMEM150C: intron variant
rs10208223	−0.0248463	0.00603189	3.94 × 10^−5^	LOC730100: intron variant
rs1051104	−0.0300326	0.00604692	7.34 × 10^−7^	SLC6A12: intron variant
rs10944	−0.0250323	0.00544036	4.44 × 10^−6^	BHMT2: 3′-UTR variant
rs11062011	−0.0331439	0.00561343	4.10 × 10^−9^	SLC6A12
rs12208357	−0.0795363	0.01839576	1.60 × 10^−5^	SLC22A1: missense variant LOC124901452: 2KB upstream variant
rs12566456	−0.0891763	0.02174221	4.25 × 10^−5^	KCNH1: intron variant
rs12827	0.08231739	0.02003506	4.13 × 10^−5^	IQSEC3-AS1: intron variant IQSEC3: 3′-UTR variant
rs13088151	−0.0340056	0.00806313	2.57 × 10^−5^	CNTN4: intron variant
rs141112	0.03233318	0.00772166	2.93 × 10^−5^	HLA-DOA HLA-DPA1
rs1644004	−0.0248609	0.00551393	6.87 × 10^−6^	DMGDH: intron variant
rs16965026	−0.0507124	0.01204763	2.66 × 10^−5^	LOC105370772: intron variant
rs17692962	−0.0316486	0.00573103	3.74 × 10^−8^	IQSEC3: intron variant IQSEC3-AS1: intron variant
rs183355	−0.0250172	0.00568667	1.14 × 10^−5^	SLC6A12: 3′-UTR variant IQSEC3-AS1: 2KB upstream variant
rs185077	−0.0238111	0.0054365	1.24 × 10^−5^	DMGDH: intron variant
rs1902623	−0.0534341	0.01237439	1.64 × 10^−5^	RPS1
rs1903530	−0.0332367	0.00770002	1.66 × 10^−5^	RNA5SP173
rs1986680	−0.0394049	0.00961203	4.29 × 10^−5^	SYT16
rs2063345	0.02946126	0.00698375	2.56 × 10^−5^	SLC22A3: intron variant
rs2075228	−0.0305374	0.00629958	1.34 × 10^−6^	SLC6A12: intron variant
rs216244	0.02198786	0.0052052	2.50 × 10^−5^	SLC6A12: intron variant
rs2174914	0.02927708	0.00702309	3.18 × 10^−5^	SLC22A3: intron variant
rs2284329	0.02429431	0.00544388	8.50 × 10^−6^	SLC6A12: intron variant
rs2284330	0.02460784	0.00530129	3.66 × 10^−6^	SLC6A12: intron variant
rs2291922	−0.0435999	0.00680552	1.82 × 10^−10^	IQSEC3-AS1: noncoding transcript variant IQSEC3: intron variant
rs233162	−0.0269973	0.00663008	4.83 × 10^−5^	ZNF385D: intron variant
rs2457575	0.02982937	0.00698443	2.03 × 10^−5^	SLC22A3: intron variant
rs2568131	−0.0590011	0.01437736	4.21 × 10^−5^	NAV2: intron variant LOC124902644: 2KB upstream variant LOC124902644: 2KB upstream variant
rs2661834	0.02939491	0.00699923	2.78 × 10^−5^	SLC22A3: intron variant
rs2702450	−0.0315948	0.00772003	4.42 × 10^−5^	RNA5SP173
rs2834932	0.02879038	0.00705378	4.63 × 10^−5^	LOC100506403: intron variant
rs2858458	0.03220939	0.00772612	3.18 × 10^−5^	HLA-DOA HLA-DPA1
rs2876711	−0.0270062	0.0057624	2.95 × 10^−6^	LOC105370265 KCTD12
rs293711	0.02357265	0.00575914	4.41 × 10^−5^	BPIFB9P
rs3128924	0.02731147	0.00636548	1.86 × 10^−5^	HLA-DPA2 COL11A2P1
rs3135021	0.03408974	0.00817301	3.15 × 10^−5^	HLA-DPA1: intron variant HLA-DPB1: intron variant
rs377551	0.02871405	0.00694405	3.68 × 10^−5^	SLC22A3: intron variant
rs379569	0.03232336	0.00772832	3.00 × 10^−5^	HLA-DOA HLA-DPA1
rs380468	0.03220939	0.00772612	3.18 × 10^−5^	HLA-DOA HLA-DPA1
rs382198	0.03233488	0.00772772	2.97 × 10^−5^	HLA-DOA HLA-DPA1
rs394487	0.02899207	0.00694306	3.09 × 10^−5^	SLC22A3: intron variant
rs412065	0.03222353	0.00772846	3.17 × 10^−5^	HLA-DOA HLA-DPA1
rs426523	0.03220939	0.00772612	3.18 × 10^−5^	HLA-DOA HLA-DPA1
rs439852	0.03220939	0.00772612	3.18 × 10^−5^	HLA-DOA HLA-DPA1
rs444821	0.03220939	0.00772612	3.18 × 10^−5^	HLA-DOA HLA-DPA1
rs495360	−0.0277466	0.00527505	1.58 × 10^−7^	SLC6A12
rs4974157	0.02405487	0.00537685	8.08 × 10^−6^	ERC2: intron variant
rs505387	−0.0300213	0.00613914	1.08 × 10^−6^	SLC6A12: 3′-UTR variant
rs510714	−0.0257809	0.00595824	1.58 × 10^−5^	SLC6A12: intron variant
rs542736	−0.0291588	0.0058391	6.39 × 10^−7^	SLC6A12: intron variant
rs557881	−0.0281208	0.00517767	6.22 × 10^−8^	SLC6A12: missense variant
rs562487	−0.0247541	0.00535511	4.01 × 10^−6^	BHMT: 2KB upstream variant
rs569919	0.02760991	0.0067914	4.96 × 10^−5^	SLC22A3
rs570680	−0.0338119	0.00559611	1.79 × 10^−9^	SLC6A13: intron variant
rs579292	−0.0226518	0.00530637	2.05 × 10^−5^	LOC105371956: intron variant
rs586199	−0.0264621	0.00548411	1.49 × 10^−6^	BHMT
rs614564	0.02621931	0.00624998	2.84 × 10^−5^	LOC105378088: 2KB upstream variant
rs631151	−0.0253154	0.00552072	4.78 × 10^−6^	DMGDH: intron variant
rs633159	−0.0289333	0.00590165	1.02 × 10^−6^	SLC6A12: 3′-UTR variant
rs6457709	0.03100779	0.00739251	2.84 × 10^−5^	HLA-DPA1
rs663310	0.03222585	0.0077285	3.17 × 10^−5^	HLA-DOA HLA-DPA1
rs7015522	0.04962682	0.01045299	2.19 × 10^−6^	CARSIP2
rs7171449	−0.0502894	0.01103103	5.43 × 10^−6^	LOC105370772: intron variant
rs7599255	−0.0301309	0.0072983	3.79 × 10^−5^	ALK: intron variant
rs7613818	0.02124971	0.00522684	4.96 × 10^−5^	ERC2: intron variant
rs7782195	0.02406508	0.0056471	2.12 × 10^−5^	MAGI2: intron variant
rs7960096	−0.025664	0.00575139	8.52 × 10^−6^	IQSEC3-AS1: intron variant IQSEC3: 3′-UTR variant
rs7971499	−0.0223223	0.00513152	1.42 × 10^−5^	SLC6A12: 3′-UTR variant
rs8093227	−0.031431	0.00719859	1.32 × 10^−5^	LOC105371956: intron variant
rs9293761	0.02368349	0.00547998	1.62 × 10^−5^	ARSB
rs9873480	0.02583322	0.00609977	2.38 × 10^−5^	ERC2: intron variant

Model 3 adjusted for age, sex, and PCs.

**Table 4:** Serum DMG model results adjusted for age, sex, and PCs.

SNP	Log (β)	SE	*p*-Value	Gene
rs10043368	−0.0163339	0.004018	4.97 × 10^−5^	ZNF366: intron variant
rs1022954	0.02230921	0.00541474	3.93 × 10^−5^	MYO16: intron variant
rs10514154	−0.0362616	0.00824616	1.15 × 10^−5^	DMGDH: intron variant
rs1055749	0.02127731	0.0048998	1.47 × 10^−5^	MCPH1-AS1: intron variant MCPH1: 3′-UTR variant
rs1057091	0.02250277	0.00490949	4.83 × 10^−6^	MCPH1: missense variant MCPH1-AS1: intron variant
rs11252395	−0.0238639	0.00517109	4.16 × 10^−6^	LOC105376368
rs11996743	−0.0157811	0.00385836	4.47 × 10^−5^	ZFPM2-AS1: intron variant
rs12211424	−0.0381197	0.00851944	8.05 × 10^−6^	ZNF184: intron variant
rs1385961	−0.0368556	0.00818561	7.07 × 10^−6^	RNU6-214P
rs1385962	−0.0360201	0.00813816	1.01 × 10^−5^	RNU6-214P
rs1422096	−0.026488	0.00628629	2.61 × 10^−5^	LOC105379107: intron variant
rs16876302	−0.0280152	0.00587175	1.95 × 10^−6^	DMGDH: intron variant
rs17054397	0.04219231	0.01032575	4.54 × 10^−5^	ITK: 3′-UTR variant
rs17082203	−0.0343162	0.00814404	2.61 × 10^−5^	RNU6-214P
rs17082229	−0.0344952	0.00814271	2.37 × 10^−5^	RNU6-214P
rs17155846	−0.0274697	0.00628538	1.30 × 10^−5^	LOC105379107: intron variant
rs17551120	0.03625213	0.00799345	6.06 × 10^−6^	STXBP5-AS1: intron variant
rs1805072	−0.0373494	0.00824437	6.21 × 10^−6^	DMGDH: synonymous variant
rs1805074	−0.0278176	0.0058738	2.32 × 10^−6^	DMGDH: missense variant
rs1872624	−0.0158555	0.00383831	3.75 × 10^−5^	B4GALT7: intron variant
rs1998084	0.01772614	0.00411422	1.72 × 10^−5^	LOC100505664: intron variant
rs2034900	−0.0281137	0.00587331	1.81 × 10^−6^	DMGDH: intron variant
rs2431332	0.03143612	0.00567521	3.40 × 10^−8^	DMGDH: intron variant
rs2433149	0.02181202	0.00490801	9.27 × 10^−6^	MCPH1-AS1: intron variant MCPH1: 3′-UTR variant
rs250513	0.02676484	0.00618679	1.59 × 10^−5^	DMGDH: intron variant
rs28326	0.03198695	0.00733647	1.36 × 10^−5^	DMGDH: intron variant
rs3758562	0.02019445	0.00488116	3.65 × 10^−5^	PRF1: intron variant
rs3792849	−0.0178398	0.00427434	3.11 × 10^−5^	B4GALT7: intron variant
rs3892245	0.04203865	0.01032946	4.87 × 10^−5^	ITK: 3′-UTR variant
rs4365962	−0.0370252	0.00818995	6.49 × 10^−6^	RNU6-214P
rs4421087	0.02065935	0.00451312	4.97 × 10^−6^	SNP not documented in dbGaP
rs4458584	−0.0161164	0.00384608	2.90 × 10^−5^	B4GALT7
rs4522	−0.0175608	0.00395074	9.23 × 10^−6^	HSBP1: 3′-UTR variant
rs4685654	−0.0295262	0.00651226	6.10 × 10^−6^	LRRN1
rs4750458	0.03575054	0.00826126	1.58 × 10^−5^	FRMD4A: intron variant
rs4891832	−0.0223609	0.00518445	1.68 × 10^−5^	LOC105372180
rs506500	0.02136081	0.00460962	3.80 × 10^−6^	BHMT: intron variant LOC124901012: intron variant
rs585800	0.02595743	0.00498842	2.14 × 10^−7^	BHMT: 3′-UTR variant
rs6044680	−0.0179242	0.0041538	1.67 × 10^−5^	RNU1-131P
rs631305	0.04246729	0.00716996	3.66 × 10^−9^	BHMT
rs642431	0.039844	0.00640411	5.88 × 10^−10^	DMGDH: intron variant BHMT2: 2KB upstream variant
rs642934	0.039844	0.00640411	5.88 × 10^−10^	DMGDH: intron variant BHMT2: 2KB upstream variant
rs6774615	−0.0302061	0.0069094	1.29 × 10^−5^	SLC6A6: intron variant
rs6796254	0.02049116	0.0050114	4.49 × 10^−5^	LOC105376959
rs694290	0.02072496	0.00461362	7.42 × 10^−6^	BHMT: intron variant
rs7217560	0.02483543	0.00528401	2.76 × 10^−6^	COPS3
rs7220577	0.02080798	0.0049688	2.93 × 10^−5^	COPS3
rs731124	−0.0275095	0.005856	2.79 × 10^−6^	DMGDH: noncoding transcript variant
rs7934785	−0.0188417	0.00412737	5.27 × 10^−6^	CNTN5: intron variant
rs8050249	−0.0162909	0.00400241	4.86 × 10^−5^	HS3ST4: intron variant
rs8134775	0.0272707	0.00600288	5.85 × 10^−6^	LINC00310
rs9301307	0.01529968	0.00372773	4.20 × 10^−5^	MYO16: intron variant
rs933684	−0.0279284	0.00587222	2.10 × 10^−6^	DMGDH: intron variant
rs9489944	−0.0301432	0.00737781	4.55 × 10^−5^	RNU6-214P

Model 3 adjusted for age, sex, and PCs.

**Table 5:** GWAS results for SNPs classically associated with choline and folate metabolism (model 3).

Gene	SNP	Log (β)	SE	*p*-Value
MTHFR	rs1801133	−0.00587	0.00397	0.139
	rs1801131	0.00172	0.00415	0.679
MTHFD1	rs2236225	NA	NA	NA
RFC	rs1051266	−0.00683	0.00344	0.0469
MTR	rs1805087	0.00192	0.00560	0.731
MTRR	rs1801394	−0.00250	0.00338	0.460
CHKA	rs10791957	0.00365	0.00340	0.284
CHDH	rs9001	NA	NA	NA
	rs12676	NA	NA	NA
BHMT	rs3733890	0.00514	0.00469	0.274
PEMT	rs12325817	NA	NA	NA
	rs4646343	NA	NA	NA
	rs2266782	−0.00102	0.00351	0.772
SLC44A1	rs7873937	NA	NA	NA
	rs3199966	0.00182	0.00957	0.849

Abbreviation: NA, not available. Model 3 adjusted for age, sex, and PCs.

**Table 6:** GWAS of serum choline-to-betaine ratio (model 3).

SNP	β	SE	*p*-Value	Gene
rs6681578	0.00991093	0.00236933	2.99 × 10^−5^	CACHD1: intron variant
rs2164720	0.01467305	0.00330247	9.31 × 10^−6^	LDAH: intron variant
rs13026309	0.00674695	0.00146867	4.60 × 10^−6^	THADA: intron variant
rs3136247	0.01103863	0.00244463	6.65 × 10^−6^	MSH6: intron variant
rs1800932	0.01074649	0.00243654	1.08 × 10^−5^	MSH6: synonymous variant
rs7711253	0.0165464	0.00354381	3.21 × 10^−6^	BDP1: intron variant
rs7707956	0.01125355	0.00271287	3.48 × 10^−5^	No documented gene consequence
rs7706073	0.01920656	0.00467906	4.20 × 10^−5^	LINC01950: intron variant
rs2288068	0.01567417	0.00376542	3.27 × 10^−5^	CYFIP2: intron variant
rs17307478	0.01523564	0.0036602	3.27 × 10^−5^	KIAA0319: intron variant
rs13215160	0.01626786	0.00388485	2.93 × 10^−5^	GABRR1: intron variant
rs12208357	0.02262707	0.00509501	9.40 × 10^−6^	SLC22A1: missense variant LOC124901452: 2KB upstream variant
rs756513	0.0067078	0.0015018	8.35 × 10^−6^	No documented gene consequence
rs17364251	0.01394374	0.00331615	2.72 × 10^−5^	No documented gene consequence
rs10818754	0.01277969	0.00295672	1.61 × 10^−5^	RC3H2: 3′-UTR variant
rs11222960	0.0269977	0.00623105	1.54 × 10^−5^	NTM: intron variant
rs2291922	0.01111452	0.00188971	4.69 × 10^−9^	IQSEC3-AS1: noncoding transcript variant IQSEC3: intron variant
rs17692962	0.00774825	0.00159149	1.20 × 10^−6^	IQSEC3: intron variant IQSEC3-AS1: intron variant
rs7960096	0.00691708	0.0015951	1.51 × 10^−5^	IQSEC3-AS1: intron variant IQSEC3: 3′-UTR variant
rs183355	0.00698278	0.00157733	1.00 × 10^−5^	SLC6A12: 3′-UTR variant IQSEC3-AS1: 2KB upstream variant
rs633159	0.00810786	0.00163617	7.77 × 10^−7^	SLC6A12: 3′-UTR variant
rs505387	0.00795451	0.00170302	3.18 × 10^−6^	SLC6A12: 3′-UTR variant
rs559759	0.00648629	0.00156154	3.40 × 10^−5^	SLC6A12: 3′-UTR variant
rs7971499	0.00660971	0.0014223	3.56 × 10^−6^	SLC6A12: 3′-UTR variant
rs1051104	0.00772504	0.00167796	4.39 × 10^−6^	SLC6A12: intron variant
rs542736	0.00768298	0.001621	2.28 × 10^−6^	SLC6A12: intron variant
rs2075228	0.00782415	0.0017485	8.04 × 10^−6^	SLC6A12: intron variant
rs2284329	−0.0065039	0.0015107	1.74 × 10^−5^	SLC6A12: intron variant
rs2284330	−0.0065495	0.0014704	8.84 × 10^−6^	SLC6A12: intron variant
rs557881	0.00668919	0.00143866	3.52 × 10^−6^	SLC6A12: missense variant
rs495360	0.0071813	0.00146364	9.96 × 10^−7^	SLC6A12
rs11062011	0.00837156	0.00156026	8.93 × 10^−8^	SLC6A12
rs570680	0.00858784	0.00155116	3.46 × 10^−8^	SLC6A13: intron variant
rs2220168	0.00789697	0.00192738	4.33 × 10^−5^	ITPR2: intron variant
rs11174335	0.00889376	0.00189291	2.78 × 10^−6^	TAFA2: intron variant
rs11174342	0.00902436	0.00195687	4.23 × 10^−6^	TAFA2: intron variant LOC124902950: intron variant
rs12578778	0.01992362	0.0047782	3.17 × 10^−5^	ANO4: intron variant
rs16934132	0.00691779	0.0015842	1.32 × 10^−5^	ACACB: intron variant
rs7976552	0.00744949	0.00163517	5.51 × 10^−6^	ACACB: intron variant
rs16947978	0.02028651	0.00453305	8.02 × 10^−6^	KSR2: intron variant
rs1681688	0.00584176	0.00142875	4.49 × 10^−5^	TMEM132D: intron variant
rs17078172	0.01505929	0.0035616	2.45 × 10^−5^	HMGA1P6
rs7335200	0.01471746	0.00353061	3.18 × 10^−5^	HMGA1P6
rs1984163	0.00950216	0.00221904	1.93 × 10^−5^	HMGA1P6
rs2876711	0.00724716	0.00159826	6.09 × 10^−6^	LOC107984587
rs10135123	0.01175498	0.00289155	4.97 × 10^−5^	URK1
rs7171449	0.01252207	0.00306117	4.46 × 10^−5^	LOC105370772: intron variant
rs1902623	0.01417481	0.00343231	3.77 × 10^−5^	RPS15P8
rs2929644	0.02090622	0.00503746	3.45 × 10^−5^	LOC102724253
rs2929647	0.02416542	0.00541238	8.42 × 10^−6^	LOC102724253
rs11540961	−0.0214329	0.0049166	1.37 × 10^−5^	NUBP2: missense variant
rs4780333	0.01057356	0.00247552	2.03 × 10^−5^	CIITA: intron variant
rs16973822	0.01613107	0.00380786	2.37 × 10^−5^	RBBP6: intron variant
rs4243134	0.01381404	0.00335306	3.93 × 10^−5^	MON1B SYCE1L
rs4569298	0.01372323	0.00335339	4.42 × 10^−5^	MON1B SYCE1L
rs8093227	0.00961646	0.00199542	1.54 × 10^−6^	LOC105371956: intron variant
rs1893459	−0.007108	0.00166218	1.98 × 10^−5^	NETO1: intron variant
rs12959392	0.00626331	0.00149733	2.99 × 10^−5^	NETO1: intron variant
rs12150965	0.01503655	0.00338257	9.21 × 10^−6^	CACNG8: 2KB upstream variant
rs13013933	−0.022755	0.00549693	3.61 × 10^−5^	GPC1: 3′-UTR variant
rs13088151	0.00945497	0.00223571	2.44 × 10^−5^	CNTN4: intron variant
rs9883103	0.00739201	0.00179233	3.86 × 10^−5^	VEPH1: intron variant LOC101928236: intron variant
rs6840205	0.00987881	0.00242079	4.65 × 10^−5^	ATP10D: intron variant
rs16851681	0.00997056	0.00241252	3.72 × 10^−5^	ATP10D: missense variant
rs11935423	0.0127963	0.00314413	4.87 × 10^−5^	TRMT10A: intron variant

Model 3 adjusted for age, sex, and PCs.

**Table 7:** GWAS of serum betaine-to-DMG ratio (model 3) PCs.

SNP	Log (β)	SE	*p*-Value	Gene
rs6425992	−0.2338471	0.05303353	1.09 × 10^−5^	FTLP18
rs1277207	0.34910275	0.08397241	3.34 × 10^−5^	AKNAD1: missense variant
rs7519217	0.32552111	0.07711205	2.53 × 10^−5^	LOC343508
rs7603744	0.67931996	0.1647434	3.87 × 10^−5^	LINC01250: intron variant
rs722889	0.81880778	0.17393026	2.66 × 10^−6^	RNA5SP94
rs1841991	0.29815476	0.05141108	7.62 × 10^−9^	ARSB: intron variant
rs1717570	0.29604386	0.05125945	8.77 × 10^−9^	ARSB: intron variant
rs9293761	0.29800874	0.05053237	4.27 × 10^−9^	ARSB: intron variant
rs185077	−0.2600591	0.04999753	2.16 × 10^−7^	DMGDH: intron variant
rs248383	−0.2586772	0.0510865	4.46 × 10^−7^	DMGDH: intron variant
rs248385	−0.2648006	0.05126639	2.62 × 10^−7^	DMGDH: synonymous variant
rs631151	−0.2904679	0.05092897	1.33 × 10^−8^	DMGDH: intron variant
rs1644004	−0.2920737	0.05088538	1.08 × 10^−8^	DMGDH: intron variant
rs642431	−0.392191	0.08315844	2.55 × 10^−6^	DMGDH: intron variant; BHMT2: 2KB upstream variant
rs642934	−0.392191	0.08315844	2.55 × 10^−6^	DMGDH: intron variant; BHMT2: 2KB upstream variant
rs10944	−0.2970196	0.05019258	3.78 × 10^−9^	BHMT2: 3′-UTR variant
rs586199	−0.30439	0.05060727	2.11 × 10^−9^	BHMY
rs631305	−0.4153324	0.09307635	8.52 × 10^−6^	BHMY
rs562487	−0.2929906	0.04940669	3.51 × 10^−9^	BHMT: 2KB upstream variant
rs506500	−0.3581445	0.0594361	1.97 × 10^−9^	BHMT: intron variant LOC124901012: intron variant
rs694290	−0.3510983	0.05949219	4.16 × 10^−9^	BHMT: intron variant
rs4421087	−0.3334988	0.05816667	1.12 × 10^−8^	SNP/gene not on file
rs585800	−0.333203	0.06462924	2.76 × 10^−7^	BHMT: 3′-UTR variant
rs1915706	0.23732281	0.0531033	8.26 × 10^−6^	BHMT JMY
rs2364594	0.25671564	0.05616074	5.12 × 10^−6^	BHMT
rs13158309	0.24662904	0.05341217	4.11 × 10^−6^	JMY BHMT
rs2121107	0.25735015	0.05615016	4.84 × 10^−6^	JMY BHMT
rs12189248	0.24216394	0.05412745	8.07 × 10^−6^	LOC124901011: intron variant LOC124900205: 500B downstream variant
rs10078815	0.24999207	0.0554529	6.88 × 10^−6^	LOC124901011: intron variant
rs2591392	0.22464765	0.05429781	3.65 × 10^−5^	JMY: intron variant
rs2591387	0.23057766	0.05567505	3.58 × 10^−5^	JMY: intron variant
rs13182512	0.22635436	0.0534241	2.36 × 10^−5^	JMY: missense variant
rs7711939	0.23409743	0.05570361	2.75 × 10^−5^	JMY: intron variant
rs10074882	0.23412197	0.05571608	2.75 × 10^−5^	JMY: intron variant
rs1062326	0.22808771	0.05438296	2.85 × 10^−5^	JMY: 3′-UTR variant LOC102724530: intron variant
rs2750473	0.33949293	0.0771035	1.12 × 10^−5^	LOC107986623: intron variant
rs2297374	0.26371872	0.05811383	5.99 × 10^−6^	SLC22A1: intron variant
rs11982432	0.37733778	0.0896363	2.66 × 10^−5^	No documented gene consequence
rs763317	−0.1991543	0.04890966	4.83 × 10^−5^	EGFR: intron variant
rs12542609	0.63969567	0.1528459	2.96 × 10^−5^	LOC100419761
rs6986672	0.48012465	0.10450138	4.59 × 10^−6^	KIF13B: missense variant
rs6981956	0.48095613	0.10448875	4.40 × 10^−6^	KIF13B: intron variant
rs2461063	−0.2420174	0.05667695	2.04 × 10^−5^	LOC107986893
rs1550734	−0.2422015	0.05408795	7.92 × 10^−6^	LOC107986893
rs7040466	−0.685511	0.16709438	4.24 × 10^−5^	JKAMPP1
rs7873941	0.47965638	0.11324424	2.37 × 10^−5^	JKAMPP1
rs16937205	0.7200178	0.17095181	2.64 × 10^−5^	NAMPTP1
rs586910	0.6629388	0.12728722	2.08 × 10^−7^	PAMR1 FJX1
rs659401	0.45442497	0.10393125	1.29 × 10^−5^	PAMR1 FJX1
rs10768154	0.45909896	0.10395536	1.05 × 10^−5^	PAMR1 FJX1
rs2291922	−0.3251766	0.06319042	2.90 × 10^−7^	IQSEC3-AS1: noncoding transcript variant IQSEC3: intron variant
rs17692962	−0.2472614	0.05314565	3.47 × 10^−6^	IQSEC3: intron variant IQSEC3-AS1: intron variant
rs633159	−0.2239166	0.05470879	4.41 × 10^−5^	SLC6A12: 3′-UTR variant
rs1051104	−0.2449588	0.05603346	1.29 × 10^−5^	SLC6A12: intron variant
rs542736	−0.2330685	0.05413321	1.74 × 10^−5^	SLC6A12: intron variant
rs2075228	−0.2602225	0.05840137	8.78 × 10^−6^	SLC6A12: intron variant
rs216244	0.20164584	0.04818403	2.97 × 10^−5^	SLC6A12: intron variant
rs557881	−0.2740004	0.04785595	1.17 × 10^−8^	SLC6A12: missense variant
rs495360	−0.2736733	0.04877753	2.27 × 10^−8^	SLC6A12
rs11062011	−0.3062373	0.05191357	4.23 × 10^−9^	SLC6A13
rs570680	−0.2973386	0.05191132	1.16 × 10^−8^	SLC6A13: intron variant
rs901510	−0.1992071	0.04841748	4.03 × 10^−5^	LOC105370224
rs7198724	0.58003513	0.13640424	2.20 × 10^−5^	ZC3H18: intron variant
rs34790908	0.29965941	0.07283552	4.03 × 10^−5^	TNFSF12: 2KB upstream variant TNFSF12-TNFSF13: 2KB upstream variant
rs4511593	0.24831263	0.05896752	2.65 × 10^−5^	TNFSF12: intron variant TNFSF12-TNFSF13: intron variant
rs4227	0.28288183	0.06812976	3.42 × 10^−5^	MPDU1: Noncoding transcript variant SOX15: 500B downstream variant
rs8073498	0.23498641	0.05318078	1.04 × 10^−5^	ATP1B2 TP53
rs7505206	0.20790143	0.05021527	3.60 × 10^−5^	LOC107985171
rs12479693	−0.2026292	0.04944385	4.32 × 10^−5^	PTPRT: intron variant LOC105372623: intron variant
rs7268202	0.50667127	0.11927352	2.25 × 10^−5^	BCAS4
rs870908	0.66192585	0.14697084	7.03 × 10^−6^	RNA5SP117
rs10048970	0.76015386	0.16784891	6.25 × 10^−6^	TAMM41: intron variant
rs1529042	0.20293812	0.04929111	3.98 × 10^−5^	GPR15
rs901884	−0.4497657	0.10136571	9.57 × 10^−6^	NLGN1: intron variant
rs11940805	0.20149032	0.04789739	2.70 × 10^−5^	LOC107986306: intron variant

Model 3 adjusted for age, sex, and PCs.

## Data Availability

All data are available at dbGaP study accession phs000789.v1.p1.

## Supplementary Materials

The following supporting information can be downloaded at https://www.mdpi.com/article/10.3390/nu17162630/s1: Table S1: Choline—B-vitamin adjusted GWAS results. Table S2: Betaine—B-vitamin adjusted GWAS results. Table S3: Dimethylglycine—B-vitamin adjusted GWAS results. Table S4: Choline-to-betaine ratio—B-vitamin adjusted GWAS results. Table S5: Betaine-to-dimethylglycine—B-vitamin adjusted GWAS results. Table S6: Choline adjusted GWAS results. Table S7: Choline adjusted-GTEx GWAS results. Table S8: Choline and B-vitamin adjusted-GTEx GWAS results. Table S9: Betaine adjusted GWAS results. Table S10: Betaine adjusted-GTEx GWAS results. Table S11: Betaine and B-vitamin adjusted-GTEx GWAS results. Table S12: Dimethylglycine adjusted GWAS results. Table S13: Dimethylglycine adjusted-GTEx GWAS results. Table S14: Dimethylglycine and B-vitamin adjusted-GTEx GWAS results. Table S15: Betaine-to-dimethylglycine ratio adjusted GWAS results. Table S16: Choline-to-betaine ratio adjusted GWAS results. Table S17: Choline-to-betaine and B-vitamin adjusted-GTEx GWAS results. Table S18: Betaine-to-dimethylglycine adjusted-GTEx GWAS results. Table S19: Betaine-to-dimethylglycine and B-vitamin adjusted-GTEx GWAS results. Figure S1: Scree plot. Figure S2: Scree-Eigen plot. Figure S3: QQ plot for choline GWAS. Figure S4: QQ plot for betaine GWAS. Figure S5: QQ plot for dimethylglycine GWAS. Figure S6: QQ plot for choline GWAS (b-vitamin adjusted). Figure S7: QQ plot for betaine GWAS (b-vitamin adjusted). Figure S8: QQ plot for dimethylglycine GWAS (b-vitamin adjusted). Figure S9: QQ plot for choline-to-betaine ratio GWAS. Figure S10: QQ plot for betaine-to-dimethylglycine ratio GWAS.

## Abbreviations

The following abbreviations are used in this manuscript:

BHMT	betaine-homocysteine methyltransferase
CSGNM	Collaborative Study of Genes, Nutrients and Metabolites
dbGaP	database of Genotypes and Phenotypes
dbSNP	database of Single Nucleotide Polymorphisms
DMG	dimethylglycine
DMGDH	dimethylglycine dehydrogenase
FDR	false discovery rate
GTEx	Genotype-Tissue Expression
GWAS	genome-wide association studies
HoloTC	holotranscobalamin
IBS	identity-by-state
LDAH	lipid droplet-associated phosphatidylserine hydrolase
MAF	minor allele frequency
MASLD	metabolic dysfunction-associated steatotic liver disease
MDS	multidimensional scaling
PC	principal component
SLC	solute carrier
SNP	single nucleotide polymorphism
TMAO	trimethylamine N-oxide
