# Supplementary figures and images for: A Genome-Wide Association Study of Circulating Serum Choline, Betaine, Dimethylglycine, and Their Ratios
Source: Nutrients. 2025 Aug 14;17(16):2630. doi: 10.3390/nu17162630 (PMC12389381; doi:10.3390/nu17162630)

Figure 1

Percentage of explained variances

60

40

20

0

Dimensions

1

2

3

4

5

6

7

8

9

10

13.7%

11.2%

10.6%

10.3%

9.9%

9.8%

9.3%

9%

8.8%

7.4%

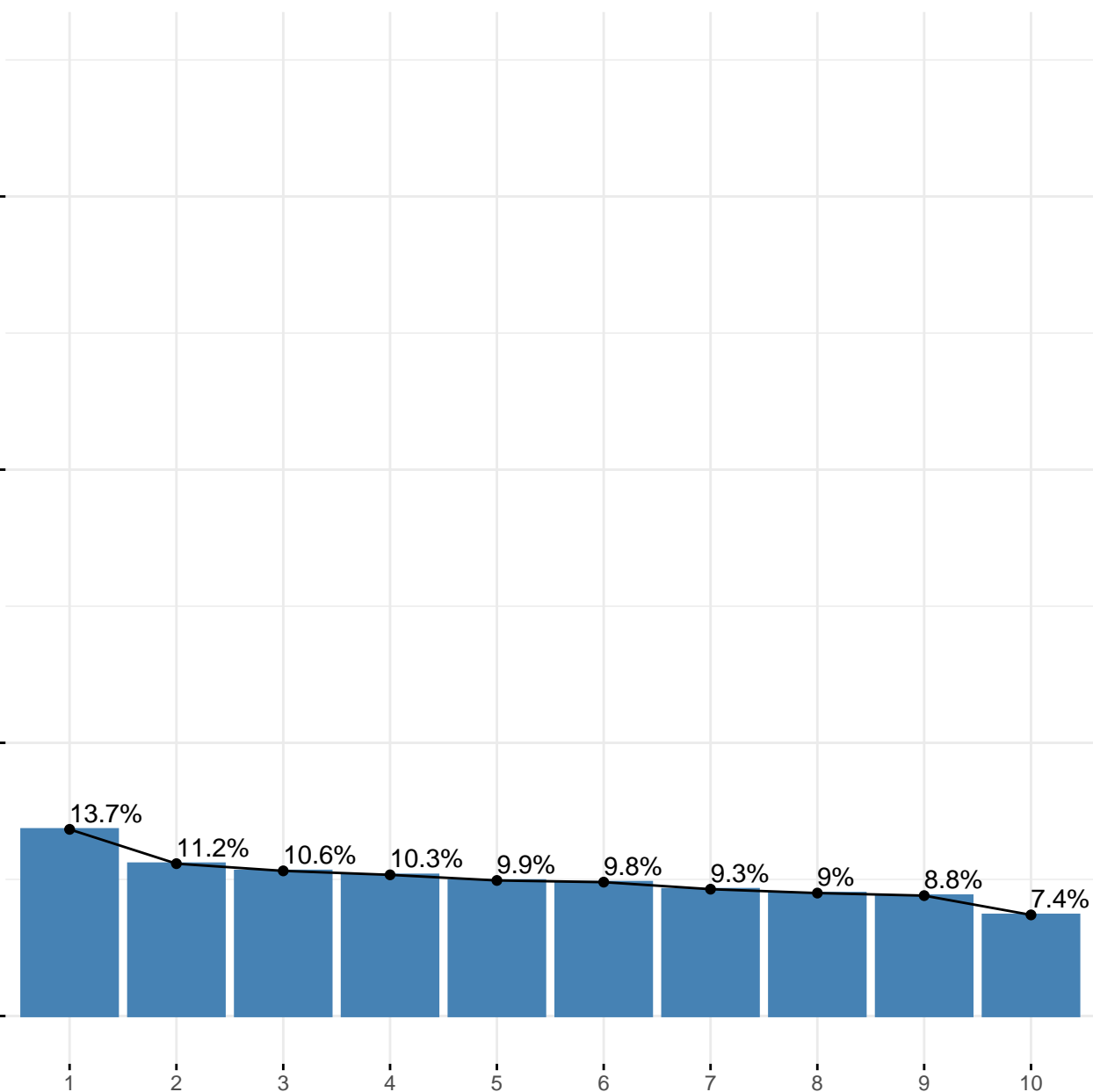

Supplement: Supplementary file 1 [file nutrients-17-02630-s001.zip › Figure S1.pdf]

Figure 2

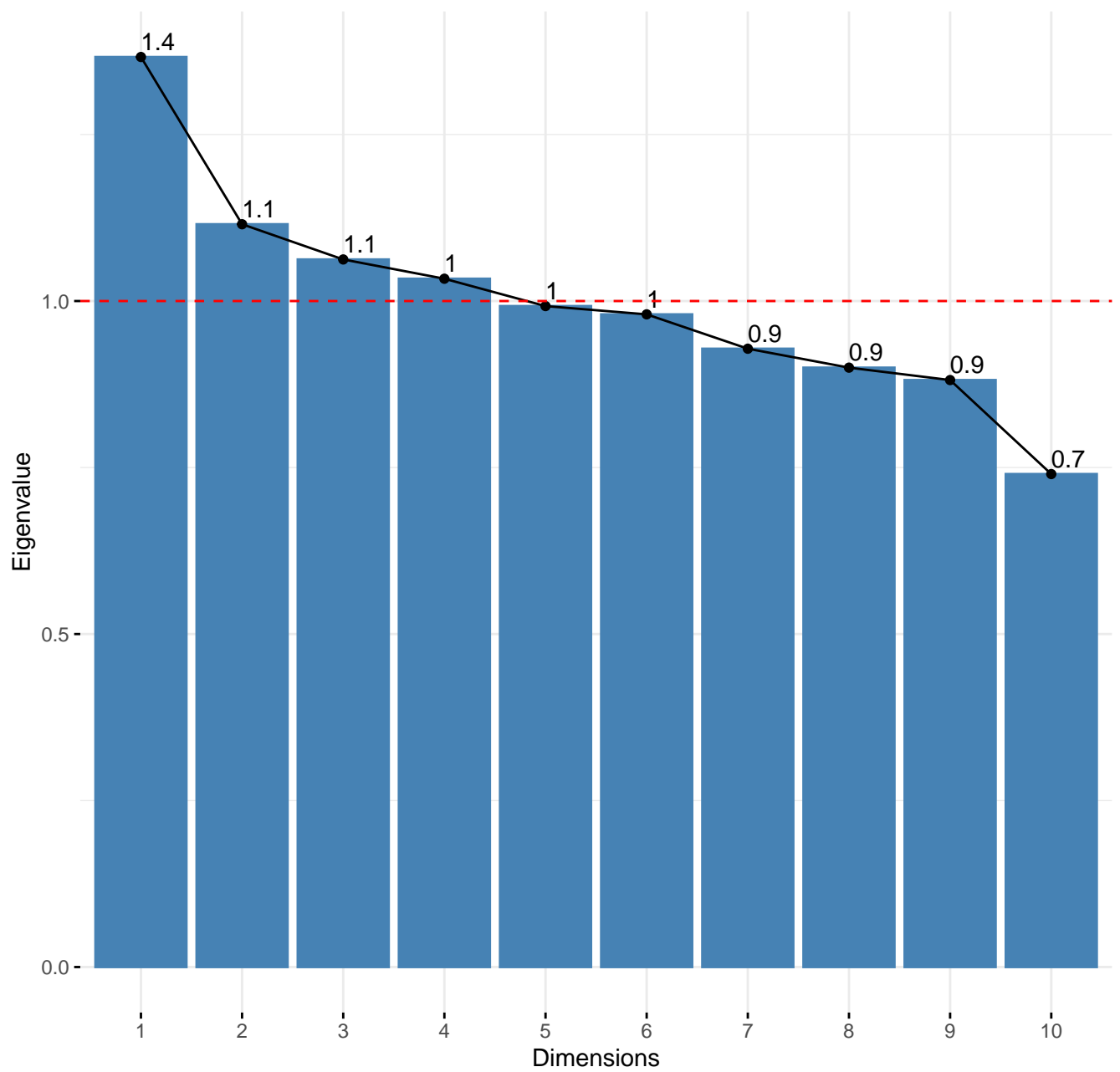

Supplement: Supplementary file 1 [file nutrients-17-02630-s001.zip › Figure S2.pdf]

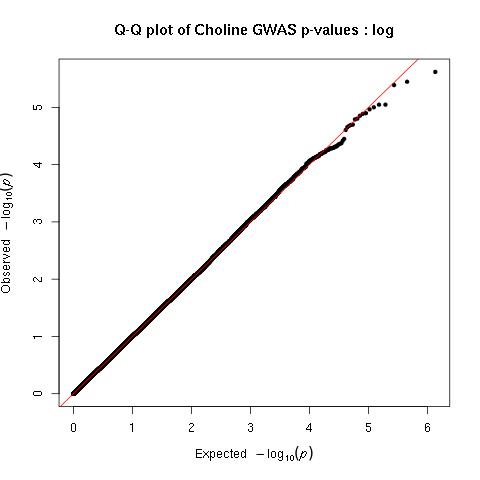

Supplement: Supplementary file 1 [file nutrients-17-02630-s001.zip › Figure S3.jpeg]

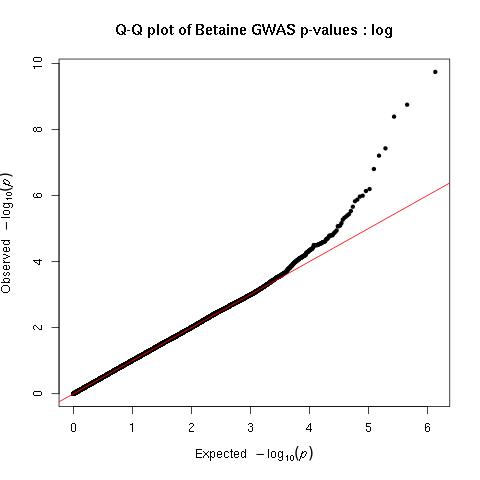

Supplement: Supplementary file 1 [file nutrients-17-02630-s001.zip › Figure S4.jpeg]

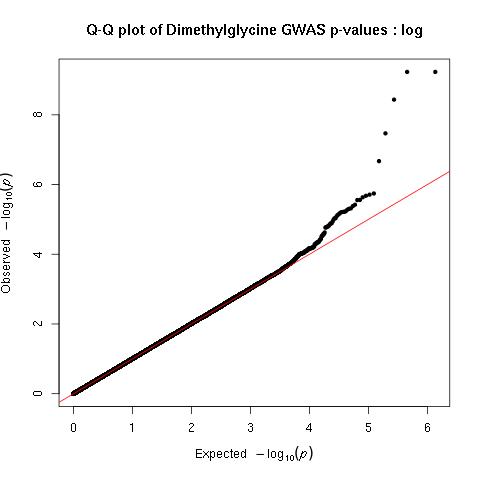

Supplement: Supplementary file 1 [file nutrients-17-02630-s001.zip › Figure S5.jpeg]

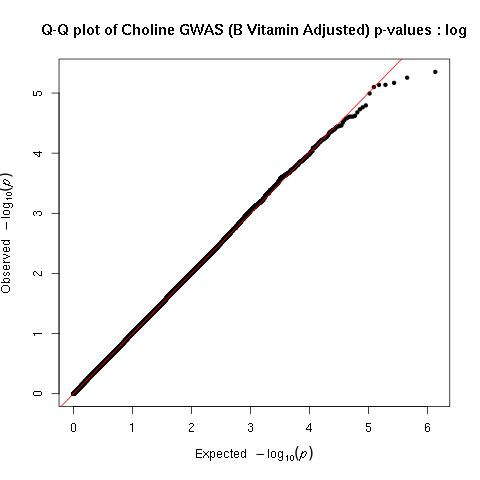

Supplement: Supplementary file 1 [file nutrients-17-02630-s001.zip › Figure S6.jpeg]

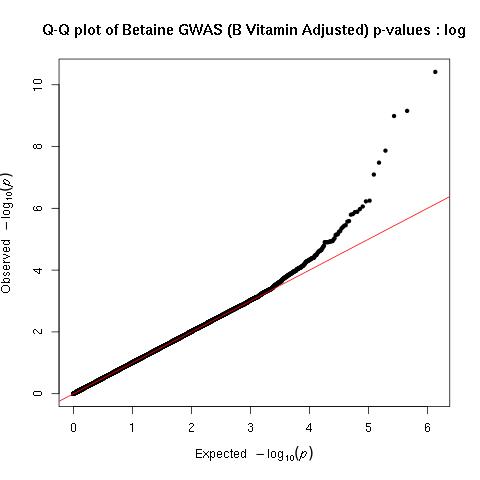

Supplement: Supplementary file 1 [file nutrients-17-02630-s001.zip › Figure S7.jpeg]

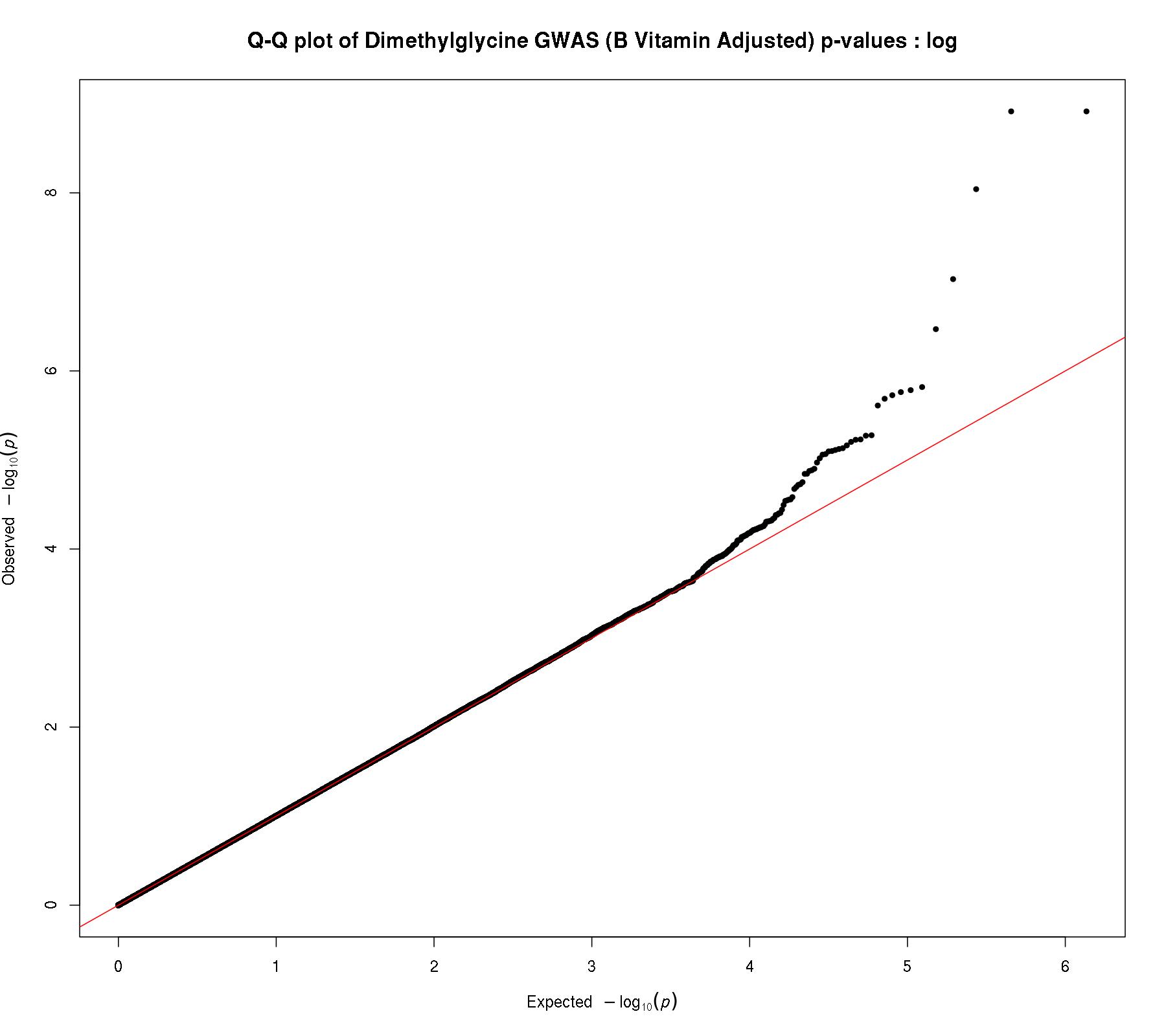

Supplement: Supplementary file 1 [file nutrients-17-02630-s001.zip › Figure S8.jpeg]

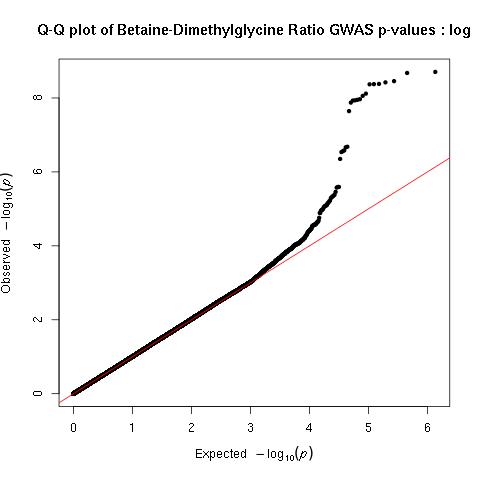

Supplement: Supplementary file 1 [file nutrients-17-02630-s001.zip › Supplementary Figure S10 QQ-Plot_bdmg.jpeg]

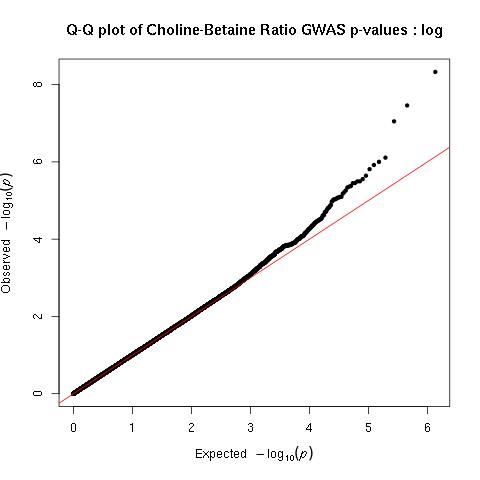

Supplement: Supplementary file 1 [file nutrients-17-02630-s001.zip › Supplementary Figure S9 QQ-Plot_cb.jpeg]
